# TCDD and Puberty in Girls

**DOI:** 10.1289/ehp.113-a17

**Published:** 2005-01

**Authors:** Mary S. Wolff, Julie A. Britton, Jose Russo

**Affiliations:** Mount Sinai School of Medicine, New York, New York, E-mail: mary.wolff@mssm.edu; Fox Chase Cancer Center, Philadelphia, Pennsylvania

We would like to comment on the article by Warner et al. (2004), in which the authors reported no significant associations between age at menarche and exposure to 2,3,7,8-tetrachlorodibenzo-p-dioxin (TCDD), an extremely potent antiestrogenic xenobiotic. The exposure of girls to TCDD at Seveso, Italy, resulted in very high serum TCDD levels (> 100 pg/g lipid), 10–100 times levels usually seen today. Warner et al. noted that the literature is mixed regarding the agonist/ antagonist effects in humans of persistent exposures of this type. First, polybrominated biphenyl exposures have been associated with earlier menarche in girls, whereas experimental models show delayed puberty, a discordance that may be due to timing of exposure (Blanck et al. 2000). Second, as Warner et al. (2004) noted, the experimental data show that TCDD and other estrogen antagonists delay vaginal opening (VO) and disrupt cyclicity in rodents treated pre-natally (Gray et al. 1997; Levy et al. 1995). However, hormonal activity depends on both timing and level of dose, such that phytoestrogens, for example, may be estrogenic—hastening VO—at high doses given after birth (Lamartiniere et al. 1995; Whitten et al. 1995).

Epidemiologic data regarding hormonally dependent female cancer are equivocal, such that there have been suggestions of a protective (i.e., antiestrogenic) effect of TCDD for breast and uterine cancer in TCDD-exposed women from Seveso (Bertazzi et al. 2001), whereas a carcinogenic effect has been observed in cohorts exposed for longer times (Manz et al. 1991; Warner et al. 2002). The findings of Warner et al. (2004), albeit not statistically significant, suggest earlier menarche with higher TCDD level among women who were younger than 8 years of age at the time of exposure [hazard ratio, 1.08 for 10-fold increase in TCDD levels; 95% confidence interval (CI), 0.89–1.30) but not among all women regardless of age. The study population appears to have the usual patterns of risk for menarche as indicated by associations that occur in the expected directions [e.g., for Seveso zone, body mass index (BMI), physical activity, alcohol intake]. Also, TCDD levels were higher among younger girls (median, 205 ppt) than in all girls (median, 140 ppt), an effect that may reflect lower BMI among younger girls and dilution of body burden by greater body size in older girls, but also a significantly higher target-organ dose.

Warner et al. (2004) examined associations in premenarcheal girls who were a younger subset (0–8 years of age) during the exposure window in 1976. This age stratum should capture any strong underlying associations among girls exposed early in life. However, it is known that pubertal transition occurs around 5–7 years of age and that age at menarche is strongly correlated with age at first signs of development (de Ridder et al. 1992; Nicolson and Hanley 1953). Therefore, hormonal exposures before 5 years of age might alter the milestones of female development, including menarche, either more potently or in a different direction than peripubertal exposures. Therefore, the youngest girls in this population (Warner et al. 2004) may have been more susceptible to hormonal effects of environmental toxicants. Recognizing the limitation of small numbers available for further age stratification, it would be interesting to know whether risk of earlier (or later) puberty was raised among girls exposed at earlier ages, such as 0–4 years.
